# Occurrence of acute infarct-like myocarditis following COVID-19 vaccination: just an accidental co-incidence or rather vaccination-associated autoimmune myocarditis?

**DOI:** 10.1007/s00392-021-01916-w

**Published:** 2021-07-31

**Authors:** Bishwas Chamling, Volker Vehof, Stefanos Drakos, Mareike Weil, Philipp Stalling, Christian Vahlhaus, Patrick Mueller, Michael Bietenbeck, Holger Reinecke, Claudia Meier, Ali Yilmaz

**Affiliations:** 1 Department of Cardiology I, Division of Cardiovascular Imaging, University Hospital Münster, Münster, Germany; 2 Department of Cardiology and Angiology, Klinikum Leer, Augustenstrasse 35-37, 26789 Leer, Germany; 3 Department of Cardiology, Electrophysiology, Gastroenterology and Endocrinology, Klinikum Vest GmbH, Dorstener Strasse 151, 45657 Recklinghausen, Germany; 4 Department of Cardiology I, Coronary and Peripheral Vascular Disease, Heart Failure, University Hospital Münster, Albert Schweitzer Campus 1, A1, 48149 Münster, Germany

**Keywords:** CMR, Myocardial infarction, Myocarditis, COVID-19 vaccination, Side effect

Sirs: the high prevalence of severe acute respiratory syndrome coronavirus-2 (SARS-CoV-2) drives us towards the urgency of vaccination to control the coronavirus disease 2019 (COVID-19) pandemic as soon as possible. Four different vaccines against COVID-19 have been authorized in the European Union (EU), following evaluation by the European Medicines Agency (EMA) so far, including two vaccines (Pfizer–BioNTech and *AstraZeneca*) that have been already used in millions of adults—and one of them (*Pfizer–BioNTech*) being recently approved even for children aged ≥ 12 years [1]. Beyond casual mild to moderate post-vaccination side effects that normally disappear within a few hours/days, some other rare adverse effects such as cerebral venous sinus thrombosis, acute ST-segment elevation myocardial infarction (STEMI) with large thrombus in coronary arteries [2] as well as (autoimmune) myocarditis [3] have also been reported recently. We would like to present the findings of three different patients that presented to our hospital until mid of June 2021 and showed unusual serious adverse cardiovascular events of infarct-like myocarditis (in the absence of CAD), possibly linked to preceding COVID-19 vaccination. CMR imaging revealed some interesting patterns of myocardial damage suggestive of “autoimmune” myocarditis that show some minor differences to the well-known CMR pattern of “viral” myocarditis.

Patient 1 was a middle-aged 68-year-old lady who initially presented to her general practitioner due to acute chest pain with radiation to her left shoulder. The rapid troponin test was positive; hence, after oral nitroglycerin administration, she was immediately referred to our emergency department. Noteworthy, this lady had been vaccinated with first dose of *AstraZeneca* the previous day. Thereafter, she had a one-time fever up to 39.0 °C, chills, and headache. On admission, a resting ECG showed an unremarkable waveform. On echocardiography, biventricular systolic function was normal and regional wall motion abnormalities could not be visualized. Laboratory parameters revealed no signs of inflammation (Supplementary Table S1). However, cardiac enzymes (high-sensitive cardiac troponin-T (hsTrop-T), creatine kinase (CK) and creatine kinase-myocardial band (CK-MB)) were elevated and showed a further significant increase within the next six hours (Fig. 1). Considering this dynamic increase and her past history with known two-vessel CAD, an immediate coronary angiography was performed, but no culprit lesion could be identified. Considering possible myocarditis after COVID-19 vaccination, a CMR study was performed on the next day (Fig. 2, upper panel): a non-ischemic pattern of myocardial damage suggestive of an active inflammatory process was observed in the basal and apical segments of the septal wall—indicating a presumably vaccination-associated autoimmune myocarditis. Noteworthy, there was no “ischemic” pattern of myocardial damage—ruling out an “ischemic” MINOCA constellation. Due to preserved systolic function and relieving symptoms, an invasive endomyocardial biopsy procedure was not indicated, and therefore not performed.

**Fig. 1:**
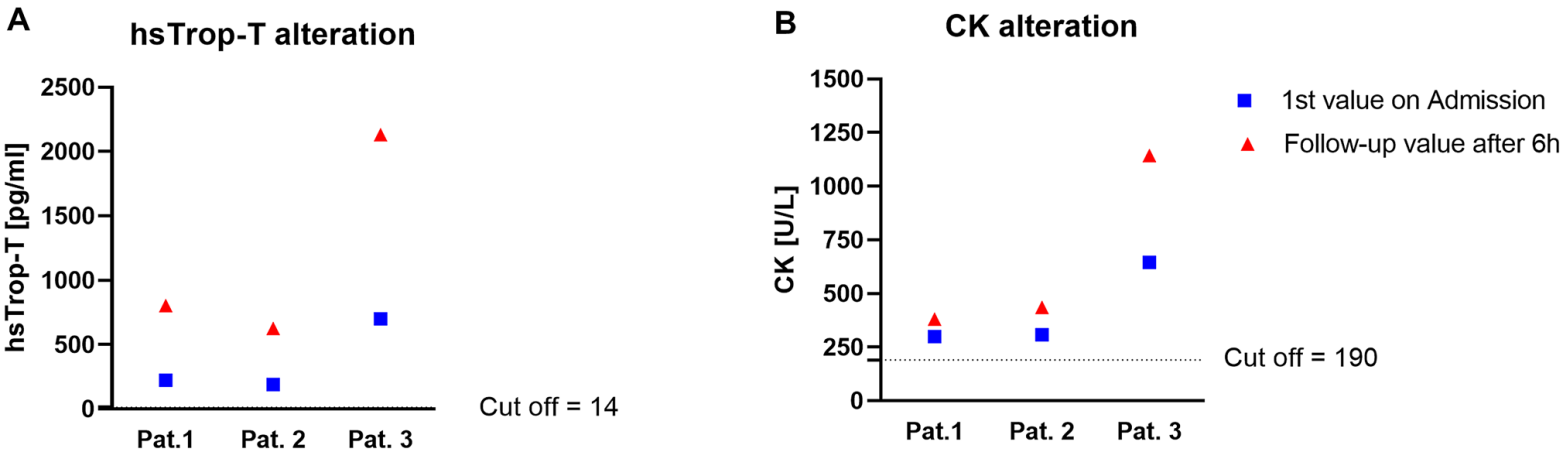
Dynamic changes of cardiac enzymes within 6 h from admission. Graph illustrating changes of cardiac enzymes within six hours after hospital admission. **A** This graph shows high-sensitive Troponin-T (hsTrop-T) values at admission and follow-up control after six hours. All patients demonstrated elevated hsTrop-T values on admission with further increase suggesting acute myocardial injury. **B** This graph illustrates creatine kinase (CK) values with a similar pattern like hsTrop-T in all patients. Cut-off values for hsTrop-T and CK are marked with thin dashed lines

**Fig. 2:**
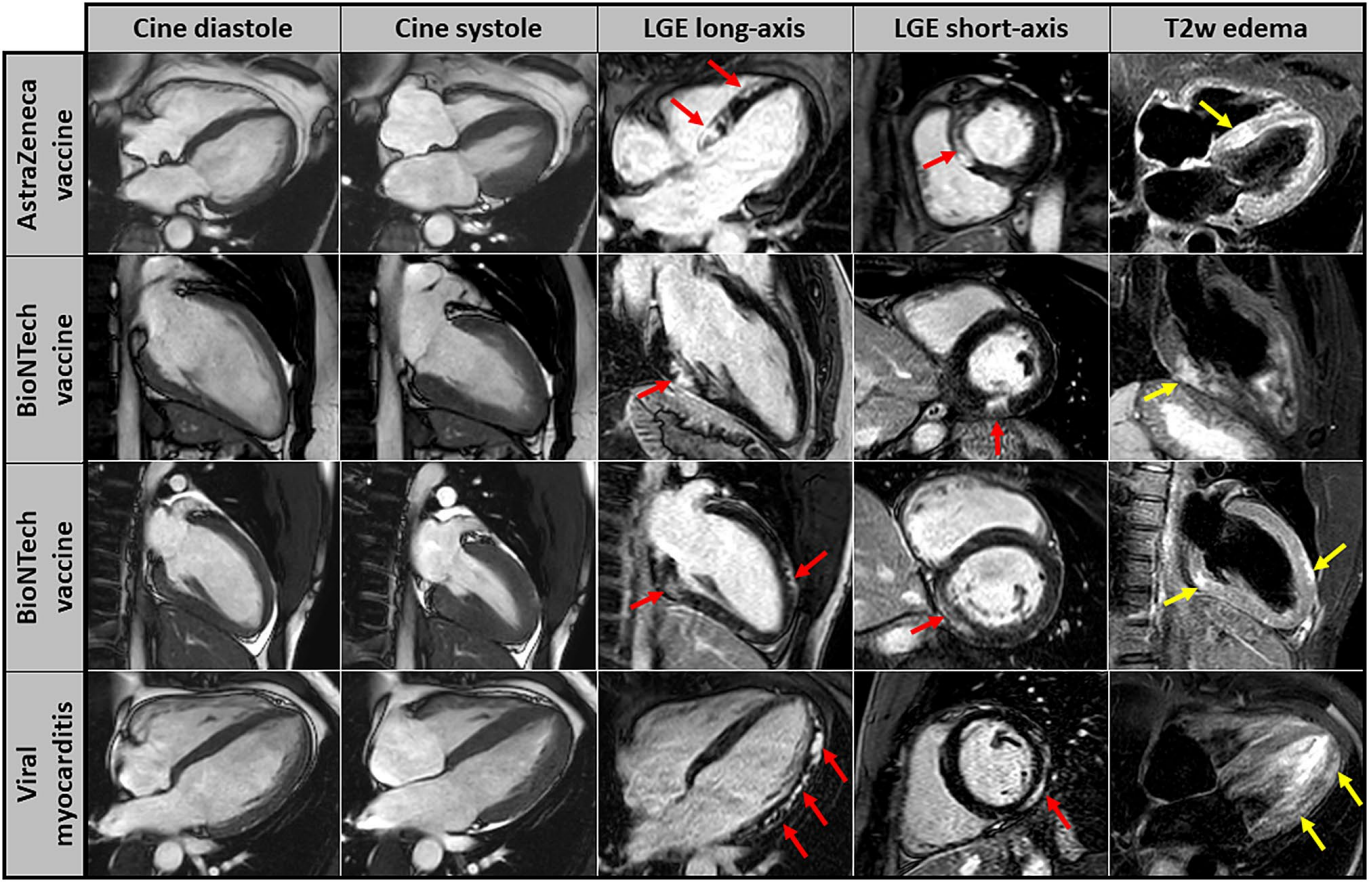
CMR images of patients showing patterns of myocardial damage after COVID-19 vaccination. Cardiovascular magnetic resonance (CMR) images including cine images at diastole and systole (first two columns), late-gadolinium-enhancement (LGE) images in long-axis and short-axis views (third and fourth column) as well as T2-weighted edema images in long-axis views (fifth column). The individual CMR-based cardiac phenotype of (a) patient 1 after 1st dose of *AstraZeneca* vaccine (upper panel), (b) patient 2 after 1st dose of *Pfizer–BioNTech* vaccine (second panel) and (c) patient 3 after 2nd dose of *Pfizer–BioNTech* vaccine (third panel) are illustrated. For comparison, the bottom panel shows CMR findings of a young patient that suffered from viral myocarditis

Patient 2, a 25-year-old young healthy man, experienced chest discomfort after getting up in the morning and informed the emergency service since his clinical symptoms persisted for hours. Therefore, he was taken to the emergency department of a local hospital with suspected acute coronary syndrome. Noteworthy, this patient had received the first dose of Pfizer–BioNTech vaccine 10 days prior to the beginning of acute chest pain. A resting ECG demonstrated inferior ST-segment elevations without other arrhythmias. After immediate application of intravenous aspirin and heparin, coronary angiography was performed, and obstructive CAD could be excluded. The left ventricle showed normal systolic function (based on both laevocardiography and echocardiography) without any signs of wall motion abnormalities. Laboratory parameters revealed no signs of inflammation (Supplementary Table S1). However, elevated hsTrop-T and CK values with further increase after six hours were suggestive of acute myocardial injury (Fig. 1). Hence, this patient was transferred to our university hospital for further diagnostic work-up including CMR that was performed on the fourth day after initial presentation (Fig. 2, second panel): CMR images revealed a non-ischemic, inflammatory focus in the basal to midventricular inferolateral wall of the left ventricle suggestive of acute myocarditis. The myocardial damage pattern (a single and rather well demarcated location) was somewhat different from the well-known pattern of viral myocarditis that occurs rather patchy and is rarely limited to one single segment (Fig. 2, bottom panel). Since there were no clinical symptoms of infection—apart from the aforementioned vaccination—prior to the beginning of acute chest pain, a presumably vaccination-associated autoimmune myocarditis was supposed in this patient.

Patient 3, a 20-year-old very healthy young police officer, presented to the emergency department of a local hospital with suspected acute coronary syndrome since he suddenly experienced a very severe chest pain. This patient reported having received a second dose of Pfizer–BioNTech vaccine three days prior to this event. Like the previous case 2, a resting ECG demonstrated ST-segment elevations in leads II, V4, V5 and V6, and significantly increased hsTrop-T as well as CK values with further dynamic increase (Fig. 1). All the other laboratory markers like CRP, leucocyte counts, and D-dimer were normal (Supplementary Table S1). Subsequent examinations including left heart catheterization and transthoracic echocardiography showed no abnormalities at all. Hence, a MINOCA constellation was suspected, and the patient referred to our hospital for further diagnostic work-up. Again, CMR imaging revealed a non-ischemic pattern of acute myocardial damage in the basal segment of the inferior LV wall and the apical segment of the anterior LV wall (Fig. 2, third panel)—suggestive of acute myocarditis. Again, a vaccination-associated autoimmune myocarditis was supposed considering the preceding COVID-19 vaccination and the pattern of myocardial damage in this patient.

More details regarding the patients’ characteristics with a major focus on CMR findings are available in Table 1 and in Table S1. Acute COVID-19 infection was ruled out in all cases based on negative SARS-CoV-2 real-time reverse transcription polymerase chain reaction (RT-PCR) tests of specimens obtained using nasopharyngeal swabs.

**Table 1:** Basic characteristics of the patients

	Patient 1	Patient 2	Patient 3
Age (years)	68	25	20
Sex	Female	Male	Male
BMI (kg/m^2^)	28.3	26.3	25.1
Clinical presentation	Acute chest pain, CCS IV	Acute chest pain, CCS IV	Acute chest pain, CCS IV
Diagnosis	Non-STEMI	STEMI	STEMI
Major ECG findings	No ST elevation	ST elevation in II, III, aVF	ST elevation in II, III, avF
COVID-19 vaccination	1st dose of *AstraZeneca*	1st dose of *Pfizer–BioNTech*	2nd dose of *Pfizer–BioNTech*
Time from vaccination to admission (days)	1	10	3
Time from event to CMR (days)	5	6	4
SARS-COV-2 (PCR)	Negative	Negative	Negative

All characteristics are from the day of admission if not mentioned otherwise

*BMI* body mass index, *CCS* angina score according to Canadian Cardiovascular Society; *STEMI* ST-segment elevation myocardial infarction, *PCR* polymerase chain reaction, *CMR* cardiovascular magnetic resonance

Numerous cases of peri- and/or myocarditis associated with various COVID-19 vaccines, as collected in the United States vaccine adverse effect reporting system (VAERS) [4] as well in the European database (EudraVigilance) [5], have been described so far. To analyze the prevalence of peri-/myocarditis with elevated troponin after *Pfizer–BioNTech* vaccination, a prospective study has already been initiated [6]. However, to the best of our knowledge, no post-vaccination cases of infarct-like autoimmune myocarditis have been reported so far illustrating a non-ischemic MINOCA constellation based on multi-parametric CMR images.

Till mid of June 2021, there were 309 (age 18–64 years) reported cases of myocarditis associated with *Pfizer–BioNTech*, 19 (age 65–85 years) with *AstraZeneca* in the EudraVigilance database [5]. Similarly, 76 and 70 cases of myocardial infarction, possibly associated with *Pfizer–BioNTech* and *AstraZeneca* vaccination, respectively, have been described in the same age group as described above [5]. Theoretically, COVID-19 vaccination may lead to an exaggerated immune response in some people and may thereby result in “autoimmune” myocardial damage—supposed to represent autoimmune myocarditis. However, it is still unclear whether there is a “direct” link between COVID-19 vaccination and myocarditis. And if so, which pathomechanisms are involved (e.g., molecular mimicry mechanism between mRNA-coded spike proteins of SARS-CoV-2 and cardiomyocyte antigens)? Moreover, we do not know whether different vaccines are associated with different immune pathways that may trigger autoimmune myocarditis and that may even determine both the frequency and severity of such an adverse effect.

Both experimental and clinical data indicate that viral infection may cause acute “viral” myocarditis and thereby also trigger chronic autoimmune processes that are involved in the pathogenesis of post-inflammatory dilative cardiomyopathy (DCM) [7]. However, detailed data regarding the association between COVID-19 vaccination and subsequent autoimmune myocarditis—with a major focus on the respective cardiac phenotype—are still missing. In a recent case report, acute infection with SARS-Cov-2 was excluded on repeated PCR tests, but a potential antigenic cross-reactivity was assumed in observation of a serological pattern that was compatible with post-vaccination immunity [8]. Another recent study reported seven cases of acute myocarditis with elevated troponin in seven male adolescents after administration of *Pfizer–BioNTech* COVID-19 vaccination, but CMR images illustrating the underlying cardiac phenotype were only given in one patient (and limited to LGE images only) [9].

The special and novel aspect of our present report is based on accurate and comprehensive cardiac phenotyping using CMR imaging in three patients presenting with an infarct-like clinical pattern following COVID-19 vaccination. Multi-parametric CMR—using amongst others robust LGE-imaging and T2-weighted edema imaging—allows to depict even subtle myocardial damages and to further differentiate between an acute inflammatory process vs. rather chronic myocardial scars [10]. Moreover, the pattern of myocardial damage visualized by CMR gives some clues regarding the underlying disease etiology as well as pathophysiology [11]. In this context, all three patients clearly demonstrated one to two LV myocardial locations of acute myocardial inflammation suggesting the presence of acute myocarditis. The respective subepicardial and/or intramural pattern (referred as non-ischemic pattern) precluded an “ischemic” origin of MINOCA in these patients. Since “viral” myocarditis is characterized by multi-focal presence of a non-ischemic LGE pattern—predominantly in the free inferolateral LV wall as well as the septum—the diagnosis of myocarditis was made in our patients. However, as illustrated in Fig. 2 (bottom panel), severe “viral” myocarditis is frequently characterized by a patchy pattern of LGE that is not only present in one to two segments. In contrast, the areas of myocardial damage in our three patients were limited and very well demarcated. Hence, these slight differences may either reflect a different severity of myocardial inflammation and/or a different underlying pathomechanism in case of COVID-19 vaccination-associated autoimmune myocarditis compared to common viral myocarditis. Future studies should, therefore, carefully consider the cardiac phenotype of myocarditis in those patients.

Importantly, the issue of potential COVID-19 vaccination-associated autoimmune myocarditis gets even more important when we consider younger people since (a) vaccination-associated adverse events were observed more frequently in younger study participants (aged < 55 years) in the respective approval studies and (b) younger people have a higher risk for immunological adverse effects due to higher reactogenicity [8]. Hence, a rapid increase in reports of post-vaccination myocarditis will—unfortunately—not be surprising based on the available data, if large vaccination campaigns will start for younger people. Therefore, regarding current discussions addressing the risk–benefit ratio of COVID-19 vaccination of children and teenagers, vaccination-associated adverse effects—including autoimmune myocarditis—need to be considered carefully, since young people are at a very low risk for severe COVID-19 infection even without vaccination.

Finally, the present report clearly indicates that we will need to consider COVID-19 vaccination-associated autoimmune myocarditis as another possible (non-ischemic) cause of MINOCA. Hence, clinicians will start to routinely ask for the timing of COVID-19 vaccination in patients presenting with a MINOCA.

We cannot definitely exclude the presence of “viral” myocarditis in our cases (making COVID-19 vaccination an innocent bystander), since we did not perform invasive endomyocardial biopsy (EMB). Although EMB still represents the gold standard for diagnosis of myocardial inflammation[12], it was not really indicated in our cases due to preserved systolic function and rather rapid improvement in clinical symptoms in each patient. The absence of preceding infectious symptoms (such as respiratory or gastrointestinal symptoms) in our patients does not exclude a “viral” cause per se but makes it rather unlikely. In case of disease progression, the collection of invasive EMB samples would be indispensable.

## Supplementary Information

Below is the link to the electronic supplementary material.

Supplementary file1 (DOCX 17 KB)

## Data Availability

Not applicable.
