# Supplementary material for: Occurrence of acute infarct-like myocarditis following COVID-19 vaccination: just an accidental co-incidence or rather vaccination-associated autoimmune myocarditis?
Source: Clin Res Cardiol. 2021 Jul 31;110(11):1850–4. doi: 10.1007/s00392-021-01916-w (PMC8325525; doi:10.1007/s00392-021-01916-w)
Supplement: Supplementary file 1 — Supplementary file1 (DOCX 17 KB) [file 392_2021_1916_MOESM1_ESM.docx]

**Supplementary Material for:**

**Chamling et al.:**  **Occurrence of acute infarct-like myocarditis following COVID-19 vaccination: just an accidental co-incidence or rather vaccination-associated autoimmune myocarditis?**

**Table S1: Additional basic characteristics of the patients**

|  | Patient 1 | Patient 2 | Patient 3 |
| --- | --- | --- | --- |
| Leucocytes [10^3^/µL]  (normal: 4-11 x 10^3^/µL) | 6.74 | 7.42 | 11.8 |
| C-reactive protein [mg/L]  (normal: <0.5 mg/dl) | <0.6 | <0.08 | 13.2 |
| Cardiovascular risk factors | ex-smoking, known CAD | smoking | none |
| Medication on admission | aspirin, β-Blocker, ACE inhibitor, statine | none | none |
| CMR findings | | | |
| LV-EF [%] | 67 | 57 | 61 |
| LVEDD [mm] | 46 | 60 | 53 |
| LV-Mass [g/m^2^] | 51 | 82 | 77 |
| RV-EF [%] | 77 | 53 | 65 |
| Late Gadolinium Enhancement (LGE) | non-ischemic pattern of myocardial damage suggestive of an active inflammatory process in the basal and apical segments of the septal wall | non-ischemic, inflammatory focus in the basal to midventricular inferolateral | non-ischemic pattern of acute myocardial damage in the basal segment of the inferior LV wall and the apical segment of the anterior LV wall |

CAD – coronary artery disease; LV-EF – left ventricular ejection fraction; LVEDD – left ventricular enddiastolic diameter, LV-Mass – left ventricular Mass
